# A Comparison Study of Quality Attributes of Ground Beef and Veal Patties and Thermal Inactivation of *Escherichia coli* O157:H7 after Double Pan-Broiling Under Dynamic Conditions

**DOI:** 10.3390/foods7010001

**Published:** 2017-12-26

**Authors:** KaWang Li, Amanda Gipe McKeith, Cangliang Shen, Russell McKeith

**Affiliations:** 1 Division of Animal and Nutritional Sciences, West Virginia University, Morgantown, WV 26506, USA; kwli@mix.wvu.edu; 2 Department of Animal Sciences & Agricultural Education, California State University Fresno, Fresno, CA 93740, USA; amckeith@mail.fresnostate.edu; 3 Division of Agriculture, College of the Sequoias, Tulare, CA 93274, USA; russellm@cos.edu

**Keywords:** *Escherichia coli* O157:H7, quality, beef, veal, thermal inactivation

## Abstract

This study compared the quality variation and thermal inactivation of *Escherichia coli* O157:H7 in non-intact beef and veal. Coarse ground beef and veal patties (2.1 cm thick, 12.4 cm diameter, 180 g) inoculated with *E. coli* O157:H7, aerobically stored before double pan-broiling for 0–360 s without rest or to 55, 62.5, 71.1, and 76 °C (internal temperature) with 0.5- or 3.5-min rest. Microbial population and qualities including color, cooking losses, pH, water activity, fat, and moisture content, were tested. After cooking the beef and veal patties, the weight losses were 17.83–29%, the pH increased from 5.53–5.60 to 5.74–6.09, the moisture content decreased from 70.53–76.02% to 62.60–67.07%, and the fat content increased (*p* < 0.05) from 2.19–6.46% to 2.92–9.45%. Cooking beef and veal samples with increasing internal temperatures decreased a* and b* values and increased the L* value. *Escherichia coli* O157:H7 was more sensitive to heat in veal compared to beef with shorter D-value and “shoulder” time. Cooking to 71.1 and 76 °C reduced *E. coli* O157:H7 by >6 log CFU/g regardless of rest time. Cooking to 55 °C and 62.5 °C with a 3.5-min rest achieved an additional 1–3 log CFU/g reduction compared to the 0.5-min rest. Results should be useful for developing risk assessment of non-intact beef and veal products.

## 1. Introduction

*Escherichia coli* O157:H7 can generate shiga toxins that can cause, with as few as 10 cells, severe hemolytic uremic syndrome in infected humans [1]. *E. coli* O157:H7 has been considered an adulterant of raw, non-intact beef products since 1999 [2]. The United States Department of Agriculture, Food Safety and Inspection Service (USDA-FSIS) defines non-intact beef products as products that have gone through treatments, such as grinding, restructuring, or mechanical tenderization processes, including cubing, needling, and pounding [3]. These non-intact beef products have been involved in several *E. coli* O157:H7 outbreaks in the United States since 2000 [4]. During non-intact beef production, pathogen cells, such as those of *E. coli* O157:H7, on the meat surface may be translocated and trapped in sterile internal tissues, thus protected from thermal destruction if the meat is undercooked. A recent survey showed that 40–58% of US consumers ordered beefsteaks at medium rare (60–62.8 °C) to rare (54.4–57.2 °C), which could potentially put consumers at a high risk from *E. coli* O157:H7 contaminated non-intact veal meat if consumers order the same way as beefsteaks [5]. 

Thermal processing includes using high temperature to inactive spoilage and foodborne pathogens is one of the most effective and widely used technologies for meat products preservation [6]. The effectiveness of cooking in inactivating *E. coli* O157:H7 contaminated non-intact beef has been documented in numerous studies [4,7,8] indicating that the cooking effectiveness on pathogen inactivation increased in the order of broiling > grilling > frying, the thicker the products the higher reduction achieved, and lower fat content increased thermal inactivation activity. 

Veal, which originated from Europe, is the meat from 16–18-week-old calves. In the past 10 years, 25% of American households purchased veal products in restaurants or retail stores at least once every three months [9]. Different veal cuts, such as cutlet, loin, rib, breast, and shank, are more popular to restaurant consumers due to their unique tenderness and flavor. Moreover, the nutrition of veal products matches the dietary guidelines that are recommended by the American Heart Association, the American Dietetic Association, and the USDA. The veal market generates approximately $1.5 billion sales each year in the US [10]. Although veal products have not been implicated in *E. coli* outbreaks in the US, since 2009 there have been multiple recalls of veal products amounting to 14,600 lb (ca. 6649 kg) due to possible *E. coli* O157:H7 and STEC contamination [11]. According to the USDA-FSIS, in May 2017, a large veal processor recalled over 5000 lb of ground veal, pork, and beef due to possible non O157:H7 shiga toxin producing *E. coli* contamination [12]. According to the USDA-FSIS, there is a greater prevalence of STEC in veal products than in other beef products. For example, in 2013, the USDA-FSIS in their testing of raw ground beef component samples in federal meat-processing factories discovered 0 (0%) of 733 samples to be positive for *E. coli* O157:H7 and three (0.24%) of 1232 samples to be positive for STEC in beef; in contrast, in veal, three (3.49%) of 86 samples were positive for *E. coli* O157:H7 and 4 (4.00%) of 100 samples were positive for STEC [13]. The difference in confirmed STEC-positive samples of veal compared to those of beef is striking and raises the question of whether the consumption of veal poses a greater risk to public health than that of beef. Currently, only two studies have reported the thermal inactivation of *E. coli* O157:H7 strains in non-intact veal products [11,14]. 

The safety of beef and veal products is important to the industry and to consumers, but consumers tend to identify the quality of products based on appearance. Cornforth and Jayasingh [15] stated that color is one of the most important characteristics regarding consumers’ purchasing decisions, even though color is sometimes poorly related to meat quality. Fresh beef or veal meat is often displayed in styrofoam trays and covered with poly-vinyl chloride (PVC) oxygen-permeable films, which allow the rapid development of the desirable bright cherry-red (beef) or light pink color (veal), respectively, due to rapid pigment oxygenation. However, discoloration often occurs within 1 week of shelf time. Currently, the number of studies that focus on the quality changes in veal products during processing, storage and cooking in terms of factors such as water activity, pH, moisture, fat content and color change is very limited. 

The objective of this study is to investigate the quality variances, including color variation in non-intact coarse ground beef and veal patties during aerobic storage and cooking and to evaluate the thermal inactivation of *E. coli* O157:H7 in coarse ground beef and veal patties. We hypothesize that (1) beef and veal patties have similar tendencies in quality change throughout storage and cooking and (2) a higher internal temperature with a longer rest time will increase the inactivation of *E. coli* O157:H7 in beef and veal patties. The novelty of this study are (1) a detailed side-by-side comparison study of quality attributes and thermal inactivation activity of *E. coli* O157:H7 between beef and veal and (2) the thermal kinetics study was conducted in a commercial size patties cooked on a griller instead of using small amount of meat heated in water bath.

## 2. Materials and Methods

### 2.1. Preparation of Bacterial Strains and Inoculum

*Escherichia coli* O157:H7 strains ATCC 43895, ATCC 43888, and ATCC 43889 (kindly provided by Beth Whittam, Michigan State University, East Lansing, MI, USA) were cultured and sub-cultured individually in 10 mL of tryptic soy broth (TSB) at 35 °C for 24 h. The three cultures were then mixed and centrifuged (Eppendorf model 5810R, Brinkmann Instruments Inc., Westbury, NY, USA) at 4629× *g* for 15 min at 4 °C. The harvested cells were washed twice with 10 mL of phosphate-buffered saline (PBS), centrifuged as described above, and re-suspended in 30 mL of fresh PBS. The washed pathogen cells were 10-fold diluted in PBS to obtain an initial inoculum level of 8 log CFU/mL, and then 40 mL of this prepared inoculum was added into 2 kg of coarse ground beef or veal to reach the inoculation level of ~6 log CFU/g. 

### 2.2. Preparation of Non-Intact Ground Veal and Beef Patties

Fresh beef knuckles and veal round top were purchased from a local meat retailer for each replicate. The meat was manually cut into trimmings and then coarse ground in a meat grinder (Gander Mountain #5 Electric Meat Grinder, Saint Paul, MN, USA) with a kidney plate (0.95 cm diameter). The ground meat was then mixed with 40 mL of the aforementioned *E. coli* O157:H7 inoculum cocktail in a bowl-lift stand mixer (Kitchen Aid Professional 600, Benton Harbor, MI, USA) at medium speed for two minutes to ensure an even distribution of the inoculum into the sample, which simulates *E. coli* O157:H7 contamination during the preparation of non-intact beef or veal products. A manual hamburger patty maker (Mainstays 6-ounce-patty maker, Walmart, Bentonville, AR) was then used to make beef or veal patties with 180 g of grounded meat. The beef/veal patties (2.1 cm thick and 12.4 cm diameter) were packaged aerobically in foam trays (20 × 25 cm, Pactiv, Lake Forest, IL, USA) with the absorbent pads, covered using air-permeable plastic film (Omni-film, Pliant Corporation, OH, USA) and stored at 4.0 °C for four days.

### 2.3. Cooking Beef or Veal Patty Samples

After four days of storage, the beef or veal patties were removed from their packages, weighed, and double pan-broiled in a Farberware grill (Farberware 4-in-1 Grill, Fairfield, CA, USA) with a set-up temperature of 177 °C (or 350 °F) (1) for 0, 30, 60, 90, 120, 180, 240, and 360 s with 0-min rest to determine the thermal dynamic parameters (i.e., D-value, “shoulder”, α) (2) to an internal geometric target temperature of 55, 62.5, 71.1, or 76 °C, followed by a 0.5- or 3.5-min rest. The cooked patties were allowed to rest on the tray after cooking without any cover. Double broiling, also known as contact grilling, is when the food (usually meat, especially burger patties, chicken, and steaks) is cooked on both sides simultaneously by applying two cooking surfaces, from both the bottom and the top, greatly reducing the cooking time. A type-K thermocouple was attached to the geometric center of the patty to monitor the internal temperature throughout cooking using PicoLog (Pico Technology Ltd., Cambridge, UK), a real-time data-recording software [4,7,8]. The cooked meat rest on the tray were also monitored the internal temperature using the same type-K thermocouple. The meat quality test including cooking losses, color, pH, water activity, moisture, and fat content were conducted in a separate study using the uninoculated beef and veal samples with the same storage and cooking treatments and cooled to room temperature after cooking.

### 2.4. Color Measurement

The objective color of non-intact beef or veal patties was measured on each day of storage and after cooking to 55, 62.5, 71.1, or 76 °C (internal and external parts) using a portable spectrophotometer (HunterLab MiniScan EZ, Reston, VA, USA), with full spectral data being obtained as L* (lightness), a* (redness), and b* (yellowness), along with reflectance data [16]. For the external surface color measurement, an average value for L*, a*, and b* was determined from the mean of three random readings on the surface from three pieces of each treatment that was used for the color analysis. To measure the internal color of the cooked samples, the beef or veal patties were split transversely across the longitudinal axis to expose the center portion with three random readings from three pieces of each treatment.

### 2.5. Physical, Chemical and Microbiological Analyses

Cooking losses were determined by measuring the difference in patty weight before cooking and after cooking when the samples had cooled to room temperature. The pH of the meat homogenate was measured after microbial analysis using a digital pH meter (Fisher Scientific, Fair Lawn, NY, USA). The water activity (aw) indicates the availability of water for bacterial growth. The water activity of the uncooked and cooked samples was measured using an AquaLab water activity meter (model series 3, Decagon Devices Inc., Pullman, WA, USA). All of the samples were tested for fat and moisture content at the Meat Science Lab of the University of Illinois at Urbana– Champaign. For microbiological analysis, the individual uncooked or cooked beef or veal samples were transferred to a Whirl-Pak filter bag (1627 mL, 19 × 30 cm, Nasco, Modesto, CA, USA) with a 1:1 ratio of nutrient broth by weight and homogenized (Masticator, IUL Instruments, Barcelona, Spain) for 2 min. Serial 10-fold dilutions of each sample in PBS were surface-plated onto tryptic soy agar (Acumedia, Lansing, MI, USA) supplemented with 0.1% sodium pyruvate (Fisher Scientific, Fair Lawn, NY; TSAP) and MacConkey agar (Acumedia, Lansing, MI, USA) for the enumeration of total bacterial populations and *E. coli* O157:H7, respectively. Colonies were counted manually after incubation at 35 °C for 48 h. The samples below the detect limit of spread-plating were enriched at 35 °C for 48 h and streak-plated onto MacConkey agar to enrich any cells that were not recovered. 

### 2.6. Data Analysis

The experiment was repeated twice, with three samples in each replicate in quality and microbial thermal inactivation studies. The quality parameters of beef and veal samples, including cooling losses, pH, water activity, fat and moisture content, were analyzed with a one-way ANOVA of SAS. All of the comparisons were performed with *p* = 0.05. Microbial populations (log CFU/g) were analyzed using the PROC MIXED procedure of Statistical Analysis System (SAS; version 9.3, SAS Institute Inc., Cary, NC, USA), with independent variables including beef or veal, cooked internal temperatures, rest time, and interactions between two or three variables. USDA-Integrated-Predictive-Modeling-Program software [17], provided by Dr. Lihan Huang, was used to estimate parameters of the survival of the pathogen cells in ground beef and veal samples during thermal processing with various heating time. The means and standard deviations were calculated, and the mean differences between treatments were determined using the Least Significant Difference (LSD) function for multiple comparisons at a significance level of α = 0.05.

## 3. Results and Discussion

### 3.1. Cooking Curve and Weight Losses

**Figure 1:**
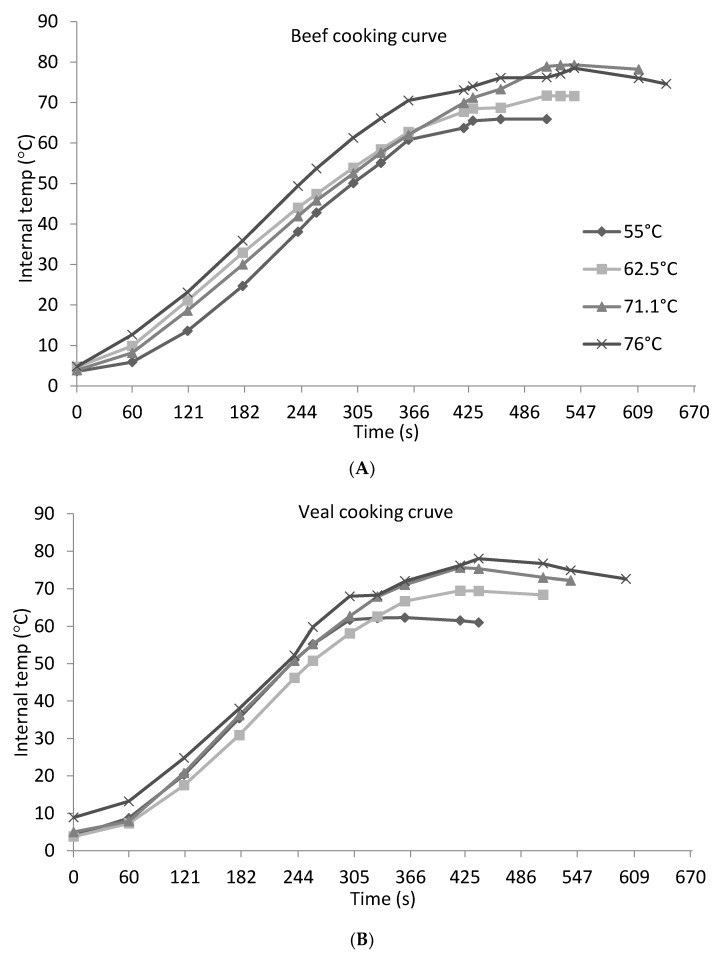
Cooking times and temperature curves for non-intact course ground beef (**A**) and veal (**B**) patties that were cooked by double pan-broiling using a Farberware grill.

The initial geometric center temperature of uncooked beef and veal patties ranged from 3.6 °C to 4.8 °C and from 4.1 °C to 8.9 °C, respectively. The cooking of beef samples by double pan-broiling required 330, 360, 430 and 460 s to reach the internal center temperatures of 55, 62.5, 71.1 and 76 °C, respectively (Figure 1A). In veal samples, it took 300, 330, 360, and 420 s to achieve internal temperatures of 55, 62.5, 71.1, and 76 °C, respectively (Figure 1B). The shorter cooking time that was required by the veal samples to reach the same internal temperatures compared to the beef samples is possibly to be explained by the following three reasons. First, the fiber density could be greater in veal than beef since veal is less mature than beef with the relatively lower muscle fiber content. Second, collagen immaturity and less fiber hypertrophy in the veal patties as compared to the more mature collagen and muscle fibers in beef muscle tissue allowing heat to transfer and penetrate the veal patties more efficiently. Third, the higher moisture content of veal could be a contributing factor to heating rate. As expected, during the 3.5-min resting time, in both beef and veal samples, the geometric center temperatures continued to increase from 61 °C to 65.9 °C, from 68.4 °C to 71.6 °C, and from 72 °C to 78.2 °C when cooking samples to 55, 62.5, and 71.1 °C, respectively (data not shown in tabular form). When cooking beef and veal samples to 76 °C, the temperature ranged from 74.6 °C to 78.5 °C and from 72.6 °C to 78 °C, respectively (data not shown in tabular form).

### 3.2. Physical and Chemical Proprieties of Beef and Veal Samples

Cooking caused weight losses ranging from 17.83% to 29% in non-intact beef samples (Table 1) and from 19% to 29% in non-intact veal samples (Table 2). In beef samples, cooking to internal temperatures of 62.5 °C to 76 °C resulted in higher (*p* < 0.05) cooking losses (24.16–29%) compared to those from cooking to 55 °C (17.83%) (Table 1). In veal samples, double pan-broiling to internal temperatures of 71.1 °C and 76 °C resulted in higher (*p* < 0.05) cooking losses (28.25–29%) (Table 2). Higher cooked internal temperature resulted in higher cooking losses due to the prolonged cooking time, causing extra moisture loss via evaporation and the release of excess juice inside the meat samples.

The pH of uncooked beef and veal patties was 5.60 (Table 1) and 5.53 (Table 2), respectively. Double pan-broiling caused a significant increase (*p* < 0.05) in the pH of beef and veal patties, resulting in pH values ranging from 5.98 to 6.09 (Table 1) and from 5.73 to 5.78 (Table 2), respectively, in agreement with the previous studies [18,19]. The increase in pH for cooked meat is due to the reduction of free acidic groups as the meat temperature increases during heating [20]. However, no significant differences in the pH of cooked samples were observed when beef or veal patties were cooked to various internal target temperatures (55 °C to 76 °C). Only a slight pH increase from 5.98 to 6.09 was detected in beef samples after cooking from 55 °C to 76 °C. 

While the moisture content describes the ratio of water mass to sample mass, water activity is the partial vapor pressure of pure water, which indicates the availability of water for bacterial growth. The water activity of fresh beef and veal patties was 0.992 (Table 1) and 0.991 (Table 2), respectively. In both beef and veal samples, the water activity did not change significantly after cooking to various internal temperatures. A previous study [21], which reported that cooking non-intact ground beef to internal temperatures of 60 °C and 65 °C resulted in water activities of 0.981 to 0.982 compared to the uncooked samples’ value of 0.982 to 0.984 had similar results to this study. The initial moisture of beef and veal samples was 70.53% and 76.02%, respectively. In both beef and veal samples, the moisture content significantly decreased (*p* < 0.05) as the cooked internal temperature increased from 55 °C to 76 °C (Table 1 and Table 2). Cooking beef or veal patties to 71.1 °C or 76 °C significantly decreased (*p* < 0.05) the moisture content to approximately 63% (beef) and 67–68% (veal) compared to the 66% (beef) and 71% (veal) in samples that were cooked to 55 °C (Table 1 and Table 2). Previous studies [4,18] reported that the moisture content of ground beef patties and of moisture-enhanced reconstructed beef patties was lower after cooking. The decreased moisture content of beef and veal is likely due to a loss of water during cooking/heating [4]. 

The fat content of fresh beef and veal patties was 6.46% and 2.19% (Table 1 and Table 2), respectively. Cooked beef samples had a significantly (*p* < 0.05) increased fat content of 8.62% to 9.45%, irrespective of the cooked internal temperatures (Table 1). Previous studies [4,19,21] reported that cooking low-fat ground beef or non-intact beef increased the fat content due to the moisture loss. A slightly (*p* = 0.074, >0.05) increased fat content ranging from 2.79% to 3.02% was found in cooked veal samples compared to that in the uncooked samples.

### 3.3. Color Variation during Storage and Cooking

The color index a*, b*, and L* values of freshly prepared beef patties were 34.75, 25.89, and 44.94, respectively (Table 3). Compared to beef samples, lower (*p* < 0.05) a* and b* values of 26.98 and 22.63 and a higher L* value of 59.83 were detected in fresh veal patties (Table 3). The less-red and lighter color is expected in veal samples because veal is the meat of bovine animals aged eight months or less, containing less myoglobin compared to the beef. During the aerobic storage, in general, the a*, b*, and L* values decreased (*p* < 0.05) from 34.75 to 15.27, from 25.86 to 13.93, and from 46.32 to 39.85 in beef patties and decreased (*p* < 0.05) from 26.98 to 12.77, from 22.63 to 16.53, and from 59.82 to 57.87 in veal samples by the end of storage. These results agree with those of a previous study [20], which found that the a*, b*, and L* values decreased in beef samples as the display time increased from 0 to three days. Madhavi and Carpenter [22] also reported that discoloration occurs within seven days of wrapping beef in oxygen-permeable film. During PVC film storage, oxymyoglobin reacted with oxygen to form metmyoglobin, causing the less-red color of the beef and veal samples. 

After cooking to 55–76 °C, the external color values of a* and b* ranged from 11.86 to 13.46 and from 16.96 to 18.45 in beef samples (Table 4(A)) and from 10.03 to 11.15 and from 18.71 to 21.53 in veal samples (Table 4(A)). In both beef and veal samples, increasing the cooked internal temperature from 55 to 76 °C did not significantly change the values of a* and b*. During double pan-broiling, the external surfaces of the beef and veal samples were in close contacted with the heat surface of the grill, which cause the oxymyoglobin to quickly become metmyoglobin, producing a brownish color regardless of the cooked internal temperature. However, it is interesting to note that the L* value of the external surface was different between beef and veal samples. Cooking beef samples from 62.5 to 76 °C decreased (*p* < 0.05) the L* value from 46.67 to 47.08 compared to the value that was obtained at 55 °C (50.32). In veal samples, the L* value decreased (*p* < 0.05) from 68.93 to 66.45 when the cooked temperature increased from 55 to 71.1 °C, while the L* value returned to 68.45 after cooking to 76 °C. There results suggest that the doneness of cooked beef and veal patties cannot be determined by external color change.

In general, the a* and b* values of the internal color of cooked beef samples decreased (less red and yellow) (Table 4(B)) and the L* value increased as the internal end-point temperature increased (Table 4(B)). For the a* and b* values, lower values of 13.95 (a*, less red) and 17.93 (b*, less yellow) were detected in the beef samples that were cooked to 76 °C compared to the 28.05 (a*) and 24.41 (b*) of the samples that were cooked to 55 °C (Table 4(B)). However, the beef samples that were cooked to 62.5 °C or 71.1 °C had similar a* values of 19.34 to 21.75, and cooking to 55 °C or 62.5 °C resulted in similar b* values of 23.51 to 24.41 (Table 4(B)). For the L* value, cooking beef samples to 62.5, 71.1 and 76 °C resulted in a higher (*p* < 0.05) value of 53.66 to 54.19 compared to the value of 50.24 in samples that were cooked to 55 °C (Table 4(B)). Hague and others [23] reported that increasing the end-point cooking temperature from 55 °C to 77 °C decreased the a* and b* values of ground beef patties from 14.6 to 11.0 and from 18.4 to 15.9, respectively, and increased the L* value from 50.9 to 52.2. The variances in the internal cooked color were attributed to the denaturation of myoglobin in ground beef patties as the internal end-point temperature increased from 55 °C to 76 °C [24]. 

Limited studies reported an internal color variation in ground veal when cooked to different end-point temperatures. In this study, a similar color variation tendency was detected in veal samples compared to that in beef samples. In cooked veal patties, cooking to an end-point temperature of 71.1 °C or 76 °C resulted in a lower (*p* < 0.05) a* value of 11.21 to 12.2 and a lower b* value of 15.56 to 16.25 than those of the samples that were cooked to 55 °C or 62.5 °C, with an a* value of 16.25 to 17.19 and a b* value of 18.49 to 18.84 (Table 4(B)). However, there was no difference (*p* > 0.05) in L* values, ranging from 70.09 to 72.96, among the veal samples that were cooked from 55 °C to 76 °C (Table 4(B)). Cooked color is important to as consumers use it in determining degree of doneness when consuming ground beef and veal. Using color exclusively could lead to the consumption of undercooked ground beef and veal, therefore, increasing the risk of foodborne illness from pathogenic bacteria.

### 3.4. Survival Curves of E. coli O157:H7 in Course Ground Beef and Veal Patties

**Figure 2:**
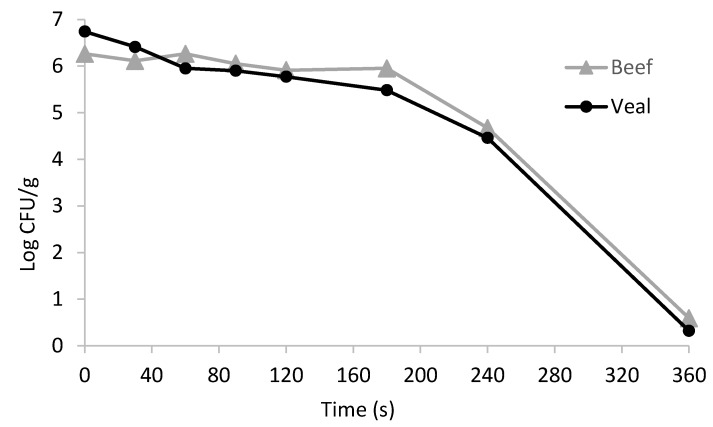
Survival curves of *Escherichia coli* O157:H7 in non-intact course ground beef and veal patties that were cooked by double pan-broiling using a Farberware grill set at 177 °C (or 350 °F).

Data points shown in the Figure 2 illustrate the survival curves of *E. coli* O157:H7 in non-intact course ground beef and veal patties after cooking at 177 °C (or 350 °F) with various heating times. As expected, the pathogen population in beef and veal samples decreased with the increasing of heating time. After cooking at 177 °C for 360 s, a reduction of 5.67 and 6.42 log CFU/g was observed in ground beef and veal samples, respectively (Figure 2). For both beef and veal samples, it was noticed that the pathogen population did not decrease significantly at the early stage (less than 120–180 s) of cooking, but when the heating time exceeded 180 s the rate of reduction started to accelerate (Figure 2). These results can be explained by the “shoulder effect” [6], which suggested that the thermal inactivation affected by the dimension of the beef and veal patties causing the geometric center temperature did not increase immediately and the pathogen located at the geometric center were not killed at the early stage. 

In this study, three survival models in the USDA-IPMP software were used to evaluate the fitness of the model to predict the thermal inactivation kinetics of *E. coli* O157:H7 cells in beef and veal samples (i.e., low value of RMSE and AIC). As shown in Table 5, the Mafart-Weibull and Buchanan Two-Phase Linear models are equally fit for describing the thermal kinetics data of beef and veal samples based on their lower RMSE (0.212 to 0.223 for beef and 0.436 to 0.516 for veal) and lower AIC scores (−67.706 to −65.257 for beef and −24.960 to −33.039 for veal) compared to the Linear model. The similar α value of beef (3.45) and veal (2.77) samples obtained from the Mafart-Weibull model indicated that the pathogen survival curves of beef and veal are in the same shape and exist an obvious “shoulder” effect [6,25] (Figure 2). 

Previous studies [26,27] has reported the D-value of *E. coli* O157:H7 in ground beef, turkey, lamb, and pork meat, with cooked temperatures from 55 °C to 65 °C in water bath settings, and no studies have reported thermal dynamic parameters of veal products yet. Results of this study showed that the D-value of *E. coli* O157:H7 in ground veal samples cooked at 177 °C on a commercial double pan-broiling griller were 63.27, 189.5, and 29.41 s calculated from Linear, Mafart-Weibull, and Buchanan Two-Phase Linear models, respectively, which were significantly lower than those from the beef samples (Table 6). According to the Buchanan Two-Phase Linear model, the shoulder time of veal is significantly lower than that of the beef samples (167.33 vs 198.19 s, Table 6). These results indicated that *E. coli* O157:H7 cells in veal samples were more sensitive to heat compared to the beef samples. 

### 3.5. Cooking Inactivation of E. coli O157:H7 Populations with Various Target Temperature and Rest Time

Before cooking, the initial *E. coli* O157:H7 population in uncooked coarse ground beef and veal samples was ranged from 6.4 to 6.6 log CFU/g (Table 7). In general, the total bacterial population counts on TSAP were similar to those that were observed on MacConkey agar in the majority of treatments, indicating that the major colonies that were found on TSAP were *E. coli* O157:H7. However, the recovery of bacterial populations on MacConkey agar was lower than that on TSAP when the veal samples were cooked to 55 °C with a 3.5-min rest and cooked to 62.5 °C with 0.5 min rest. This can be explained by the heat-injured *E. coli* O157:H7 cells not being recovered on MacConkey agar as a selective medium. 

Very limited studies have reported the thermal inactivation of *E. coli* O157:H7 in non-intact veal products. Luchansky and others [11] recently found that cooking breaded or un-breaded veal cutlets for 1.5 min per side on an electronic skillet at 191.5 °C achieved an internal temperature of 71.1 °C and a >5.0 log reduction. In a recent study of the same research group, the authors reported that cooking breaded veal cordon bleu at 191.5 °C in pre-heated extra virgin olive oil for ≤6 min or for seven to 10 min per side achieved 1.5 or 3.5 log CFU/g and ≥6.2 log CFU/g, respectively [14]. In this study, we compared the thermal-sensitive *E. coli* O157:H7 in non-intact course ground beef and veal patties. The results of this study indicate that in both beef and veal samples, the higher the cooked internal temperature, the longer the rest time, the greater reductions of *E. coli* O157:H7 was reached, and *E. coli* O157:H7 cells were more (*p* < 0.05) sensitive to heat in veal samples than in beef samples. 

As expected, double pan-broiling beef and veal samples to 71.1 °C (well done doneness) and 76 °C (beyond well-done doneness) decreased the overall pathogen populations to below the detection limit (>6 log CFU/g reduction), regardless of the rest time (Table 7), in agreement with the study [28], who reported that cooking refrigerated ground beef patties to internal temperature of 71.1 °C and 76.6 °C reduced *E. coli* O157:H7 to 5.1–7.0 log CFU/g. To reduce the possibility of food-borne outbreaks due to *E. coli* O157:H7 contamination, the USDA-FSIS recommends cooking non-intact veal products to an internal temperature of 62.5 °C (145 °F) with at least a three-minute rest time [29]. To the best of our knowledge, no research publication has reported the impact of rest time on the thermal inactivation activity of *E. coli* O157:H7 on non-intact beef and veal products. By double pan-broiling to 55 °C and 62.5 °C with a 0.5-min rest, 1.95 to 4.79 log CFU/g and 1.97 to >6.0 log CFU/g reductions of *E. coli* O157:H7 were achieved in beef and veal samples, respectively (Table 7). For beef samples, an additional (*p* < 0.05) 0.98 to 1.14 log CFU/g reduction was reached when the rest time extended from 0.5 min to 3.5 min after cooking from 55 °C to 62.5 °C (Table 7). This enhancement of thermal inactivation with a longer rest time was mainly due to the extended heating of beef and veal, causing the internal temperature to continue to increase, even after the patties were removed from the grill. Similar to beef samples, when course ground veal patties were cooked to 55 °C with a 3.5-min rest, an additional (*p* < 0.05) reduction of 2.68 log CFU/g was achieved compared to the 0.5-min rest (Table 7). It is interesting to note that the amount of surviving *E. coli* O157:H7 was below the detect limit (>6.0 log CFU/g reduction) when veal samples were cooked to 62.5 °C with a 0.5- or 3.5-min rest (Table 7), significantly (*p* < 0.05) lower than the same amount in beef samples on MacConkey agar. This result might be explained by the higher moisture content (slightly less fat) in combination with less mature and less thick collagen in veal muscle tissue, allowing for more efficient heat transfer. The compaction density of the physiologically less mature veal tissue versus beef tissue in the patty could also be a factor affecting heat transfer, which produces a greater steaming effect inside the veal patties, resulting greater pathogen reduction. Over all, the present study demonstrates that cooking coarse ground beef and veal patties to an internal end-point temperature of ≥62.5 °C with at least a 3-min rest achieves a >5.0 log reduction of *E. coli* O157:H7 cells. 

## 4. Conclusions

In conclusion, *E. coli* O157:H7 is more vulnerable in veal compared to the beef during thermal processing. A higher internal temperature and longer rest time cause an increased inactivation of *E. coli* O157:H7, and veal and beef patties present similar tendencies in terms of quality change throughout storage and cooking, supporting our hypothesis. The results of this study cover various aspects of beef and veal quality changes during storage and cooking that will be beneficial for intact and non-intact beef and veal preparation at multiple points, including retail, foodservices, and at home. It was also verified that cooking coarse ground beef or veal to an internal end-point temperature of 62.5 °C with a 3.5-min rest will not generate a great food safety risk. This information will be useful for the USDA-FSIS in developing risk assessments of *E. coli* O157:H7 in non-intact and intact beef and veal products.

## Figures and Tables

**Table 1:** Cooking losses, pH, water activity, and moisture and fat contents of non-intact course ground beef patties before and after cooking to various internal end-point temperatures.

Beef	Before Cooking	After Heating to (°C)			
		55	62.5	71.1	76
Cooking losses (%)	-	17.83 ± 5.56 ^a^	24.17 ± 2.71 ^b^	26.67 ± 2.50 ^b^	29.00 ± 1.55 ^b^
pH	5.60 ± 0.07 ^a^	5.98 ± 0.11 ^b^	6.07 ± 0.16 ^b^	6.08 ± 0.15 ^b^	6.09 ± 0.15 ^b^
Water activity	0.992 ± 0.001 ^a^	0.990 ± 0.003 ^a^	0.991 ± 0.005 ^a^	0.990 ± 0.003 ^a^	0.987 ± 0.003 ^a^
Moisture (%)	70.53 ± 0.55 ^a^	66.63 ± 1.85 ^b^	64.36 ± 1.23 ^bc^	63.04 ± 1.25 ^c^	62.60 ± 1.18 ^c^
Fat (%)	6.46 ± 0.77 ^a^	8.62 ± 0.65 ^b^	9.34 ± 0.44 ^b^	9.45 ± 0.55 ^b^	8.96 ± 0.60 ^b^

Mean values with a different lowercase letter within a row are significantly different (*p* < 0.05).

**Table 2:** Cooking losses, pH, water activity, and moisture and fat contents of non-intact course ground veal patties before and after cooking to various internal end-point temperatures.

Veal	Before Cooking	After Heating to (°C)			
		55	62.5	71.1	76
Cooking losses (%)	-	19.00 ± 3.56 ^a^	20.75 ± 5.50 ^a^	28.25 ± 0.96 ^b^	29.00 ± 0.82 ^b^
pH	5.53 ± 0.01 ^a^	5.78 ± 0.02 ^b^	5.74 ± 0.08 ^b^	5.73 ± 0.08 ^b^	5.74 ± 0.08 ^b^
Aw	0.991 ± 0.005 ^a^	0.989 ± 0.003 ^a^	0.988 ± 0.002 ^a^	0.987 ± 0.002 ^a^	0.988 ± 0.001 ^a^
Moisture (%)	76.02 ± 0.36 ^a^	71.19 ± 0.51 ^b^	69.57 ± 2.01 ^bc^	67.95 ± 0.49 ^cd^	67.07 ± 0.89 ^d^
Fat (%)	2.19 ± 0.25 ^a^	2.79 ± 0.54 ^a^	3.00 ± 0.44 ^a^	3.02 ± 0.46 ^a^	2.92 ± 0.17 ^a^

Mean values with a different letter within a row are significantly different (*p* < 0.05), Aw: water activity.

**Table 3:** Color values (L*, a*, and b*) of the external parts of course ground beef and veal patties stored aerobically at 4.0 °C for four days in foam trays that were covered with air-permeable plastic film.

	Beef					Veal				
	Day 0	Day 1	Day 2	Day 3	Day 4	Day 0	Day 1	Day 2	Day 3	Day 4
L*	46.32 ± 2.11 ^a^	42.06 ± 2.75 ^b^	40.20 ± 2.37 ^c^	43.70 ± 2.71 ^b^	39.85 ± 2.05 ^c^	59.96 ± 1.64 ^a^	59.16 ± 1.55 ^a^	58.15 ± 2.44 ^a^	58.26 ± 1.65 ^a^	57.87 ± 1.64 ^b^
a*	34.90 ± 2.27 ^a^	22.92 ± 2.61 ^b^	19.71 ± 2.26 ^c^	15.40 ± 1.61 ^d^	15.27 ± 1.02 ^d^	26.90 ± 0.94 ^a^	22.41 ± 1.38 ^b^	16.97 ± 1.49 ^c^	14.39 ± 1.04 ^c^	12.77 ± 0.60 ^d^
b*	25.86 ± 2.07 ^a^	17.91 ± 1.62 ^b^	17.14 ± 1.48 ^b^	14.40 ± 1.60 ^c^	13.93 ± 1.49 ^c^	22.61 ± 0.95 ^a^	20.07 ± 0.96 ^b^	17.99 ± 0.95 ^c^	17.30 ± 0.96 ^c^	16.53 ± 0.83 ^c^

Mean values with a different lowercase letter within a row within beef or veal are significantly different (*p* < 0.05).

**Table 4:** Color values (L*, a*, and b*) of the external (A) and internal (B) parts of course ground beef and veal patties double pan-broiling to internal end-point temperatures of 55, 62.5, 71.1, and 76 °C.

	L*		a*		b*	
Cooked Internal Temperature (°C)	Beef	Veal	Beef	Veal	Beef	Veal
**(A) External parts**						
55	50.32 ± 3.18 ^aA^	68.98 ± 2.13 ^aB^	13.46 ± 1.66 ^aA^	10.76 ± 1.59 ^abB^	18.45 ± 2.21 ^aA^	19.58 ± 2.69 ^aA^
62.5	46.87 ± 1.79 ^bA^	68.40 ± 1.08 ^aB^	12.50 ± 0.97 ^aA^	11.15 ± 0.99 ^aB^	17.33 ± 1.03 ^aA^	21.53 ± 2.78 ^bB^
71.1	47.08 ± 1.57 ^bA^	66.45 ± 1.85 ^bB^	12.63 ± 1.28 ^aA^	10.58 ± 0.64 ^abB^	17.45 ± 1.04 ^aA^	19.59 ± 1.84 ^aB^
76	46.67 ± 1.59 ^bA^	68.45 ± 1.55 ^aB^	11.86 ± 0.75 ^aA^	10.03 ± 0.53 ^bB^	16.96 ± 1.04 ^aA^	18.71 ± 2.09 ^aB^
**(B) Internal parts**						
55	50.25 ± 7.15 ^aA^	70.99 ± 3.00 ^aB^	28.06 ± 4.59 ^aA^	17.19 ± 2.43 ^aB^	24.42 ± 2.77 ^aA^	18.84 ± 1.27 ^aB^
62.5	54.20 ± 1.85 ^bA^	70.09 ± 3.32 ^aB^	21.76 ± 5.76 ^bA^	16.25 ± 3.60 ^aB^	21.31 ± 2.66 ^bA^	18.49 ± 1.79 ^aB^
71.1	53.79 ± 2.35 ^bA^	72.97 ± 2.64 ^aB^	19.34 ± 6.72 ^cA^	12.20 ± 1.15 ^bB^	20.45 ± 2.97 ^cA^	16.26 ± 0.59 ^bB^
76	53.66 ± 1.74 ^bA^	72.35 ± 2.01 ^aB^	13.95 ± 2.63 ^dA^	11.21 ± 0.97 ^bB^	17.94 ± 1.49 ^dA^	15.57 ± 0.32 ^bB^

Mean values with a different lowercase letter within a column within L*, a*, and b*are significantly different (*p* < 0.05); mean values with a different capital letter within a row within L*, a*, and b* are significantly different (*p* < 0.05).

**Table 5:** Comparison of square root of mean of sum of squared errors (RMSE) and Akaike information criterion (AIC) value for the proposed survival models on the inactivation of *E. coli* O157:H7 in ground beef and veal patties after double pan-broiling at 177 °C (350 °F) for 0 to 360 s.

Products	Index	Linear	Mafart-Weibull	Buchanan Two-Phase Linear
Beef	RMSE	0.936	0.223	0.212
	AIC	1.752	−65.257	−67.706
Veal	RMSE	0.850	0.436	0.516
	AIC	−2.908	−33.039	−24.960

**Table 6:** Parameters (mean ± standard error) of survival models estimated for the inactivation of *E. coli* O157:H7 in ground beef and veal patties after double pan-broiling at 177 °C (350 °F) for 0 to 360 s.

Model Name	Model Parameters	Beef	Veal
Linear	D	70.67 ± 8.50 ^a^	63.27 ± 6.19 ^b^
Mafart-Weibull	D	218.3 ± 7.65 ^a^	189.5 ± 14.41 ^b^
	α	3.45 ± 0.23 ^a^	2.77 ± 0.32 ^a^
Buchanan Two-Phase Linear	Shoulder	198.19 ± 5.22 ^a^	167.33 ± 10.33 ^b^
	D	33.91 ± 2.64 ^a^	29.41 ± 1.25 ^b^

Means with a different lowercase letters within a row are significantly different (*p* < 0.05).

**Table 7:** Total bacterial and *Escherichia coli* O157:H7 populations (log CFU/g; ±standard deviation) that were recovered from tryptic soy agar plus 0.1% sodium pyruvate (TSAP) and MacConkey agar, respectively, in beef and veal samples before and after double pan-broiling to 55, 62.5, 71.1, and 76 °C with a 0.5- or 3.5-min rest.

Temperature (°C)	Rest Time (min)	TSAP		MacConkey Agar	
		Beef	Veal	Beef	Veal
After inoculation	-	6.44 ± 0.05 ^aA^	6.60 ± 0.01 ^aA^	6.40 ± 0.07 ^aA^	6.40 ± 0.09 ^aA^
Before cooking	-	6.37 ± 0.65 ^aA^	6.58 ± 0.06 ^aA^	6.47 ± 0.33 ^aA^	6.35 ± 0.16 ^aA^
55	0.5	4.86 ± 0.08 ^bA^	4.32 ± 0.24 ^bA^	4.52 ± 0.29 ^bA^	3.38 ± 1.10 ^bB^
	3.5	3.32 ± 0.81 ^cA^	1.58 ± 1.28 ^cB^	3.38 ± 1.10 ^cA^	0.70 ± 0.59 ^cB^
62.5	0.5	1.87 ± 0.60 ^dA^	1.46 ± 0.54 ^cA^	1.68 ± 0.37 ^dA^	<0.3 ^dB^
	3.5	0.94 ± 0.58 ^eA^	<0.3 ^dB^	0.70 ± 0.59 ^eA^	<0.3 ^dB^
71.1	0.5	<0.3 ^fA^	<0.3 ^dA^	<0.3 ^fA^	<0.3 ^dA^
	3.5	<0.3 ^fA^	<0.3 ^dA^	<0.3 ^fA^	<0.3 ^dA^
76	0.5	<0.3 ^fA^	<0.3 ^dA^	<0.3 ^fA^	<0.3 ^dA^
	3.5	<0.3 ^fA^	<0.3 ^dA^	<0.3 ^fA^	<0.3 ^dA^

Means with a different lowercase letter within a column or a different capital letter within a row on TSAP or MacConkey agar are significantly different (*p* < 0.05).
